# The Prone-Position Whole Breast Irradiation Paradox: Where Do We Stand? A Comprehensive Review

**DOI:** 10.3390/jcm15010390

**Published:** 2026-01-05

**Authors:** Chris Monten, Ilaria Benevento, Antonietta Montagna, Edy Ippolito, Paola Anselmo, Luciana Rago, Barbara D’Andrea, Angela Solazzo, Antonella Bianculli, Raffaele Tucciariello, Giammaria Fiorentini, Vito Metallo, Simone Salvago, Carmen Santoro, Anna Vallario, Grazia Lazzari

**Affiliations:** 1 Radiation Oncology Unit, Ghent University, B-9000 Ghent, Belgium; 2 Radiation Oncology Unit, IRCCS—CROB, 85028 Rionero in Vulture, Italy; 3 Radiation Oncology Unit, Campus Biomedico, 00128 Roma, Italy; 4 Radiation Oncology Unit, Ospedale S. Maria, 05100 Terni, Italy; 5 Physic Unit, IRCCS—CROB, 85028 Rionero in Vulture, Italy; antonella.bianculli@crob.it (A.B.);; 6 Department of Oncology, San Salvatore Hospital, 61121 Pesaro, Italy; g.fiorentini2020@gmail.com

**Keywords:** breast cancer, IMRT, DIBH, whole breast irradiation, photons

## Abstract

Over the past two decades, interest in prone-position whole breast irradiation (WBI) as an effective and practical alternative to supine treatment has been growing a lot. Although solid scientific data has provided evidence of substantial dosimetric benefit with decreased toxicity, there is still conflict in the radiotherapy community over whether to adopt prone-position WBI as a valid alternative to supine radiotherapy (RT) in routine clinical practice. A large number of prone trials have been conducted to assess and address concerns related to prone treatment in large and pendulous breasts and in left and right breast cancer (BC), nodal irradiation, and its reproducibility with deep inspiration breath hold (DIBH) delivery with photons or protons. Appropriate atlases have been defined to improve prone nodal irradiation. Additionally, more comfortable customized immobilization couches have been constructed to permit IMRT beams and VMAT arrangements with modern LINACs. Although our search in literature databases shows a growing body of evidence from the past two decades on this issue, prone WBI is still underused. Given the paradox of the advances and benefits of this positioning and the lack of drive in the radiotherapy community towards its clinical implementation, the purpose of this comprehensive review is to evaluate the true advantages of this position in real life and contextualize it in scenarios like large breasts, left-sided breast cancer, and nodal irradiation to encourage its implementation in clinical practice.

## 1. Introduction

Breast cancer (BC) adjuvant strategies, encompassing advanced postoperative radiation modalities in conjunction with neoadjuvant or adjuvant novel systemic regimes, have significantly improved the long-term survival prospects of BC patients and improved BC patients’ long-term quality of life while attempting to minimize radiation-induced side effects [1,2]. The vast majority of BC patients still receive adjuvant radiotherapy for breast cancer in the supine position worldwide, although this may be challenging, mainly for BC patients showing post-surgery discomfort in terms of arm pain or arthritic arm limitations. For example, scoliosis in older women hinders the alignment of the chest on the couch with the arm positioned above the head. Several criticisms concerning the irradiation of large and pendulous breasts in obese women (because of the inflammation of the inframammary fold and axilla, owing to hotspot deposition) need to be considered [3]. In addition, concerns about the dose received by organs at risk (OARs) like the heart [4], the left anterior descending coronary artery (LADCA), and ipsilateral of lung (ILL) have impelled teams of radiation oncologists to search for practical solutions such as the use of the deep inspiration breath hold (DIBH) modality for left-sided BC [5], while alternative treatment positions in prone or lateral decubitus have also been investigated [6,7]. Prone positioning has been the most studied modality since the beginning of the 2000s, and now it is under study for use in accelerated partial breast irradiation (APBI) [8]. For a long time, this novel positioning has been hypothesized as a reasonable solution for treating pendulous breasts and reducing the dose to cardiac structures in left-sided BC [9]. Differences in prone dive and prone crawl have also been addressed as valid alternatives to the supine position in WBI, showing great advantages for many OARs in both modalities [10]. Nevertheless, there is still a paradox: there is solid scientific evidence of decreased toxicity on the one hand and a lack of drive in the radiotherapy community towards its clinical implementation on the other. In fact, when we analyze the use of prone positioning in the real world, there is scarce and patchy distribution across all countries. For example, in Geneva University Hospitals, although this positioning has been introduced into routine clinical practice since 2010, it has only been applied in a few institutions [11]. In Spain, as recorded by a survey, prone positioning has rarely been adopted, featuring in only three out of forty RT centers [12]. In German countries, only one of sixty-eight RT centers have declared that they offer heart-sparing RT in prone position [13]. In Belgium, prone WBI with RNI has been adopted by Ghent University in routine practice in conjunction with a DIBH technique in left-sided BC starting ten years ago [14]. In Italy, prone positioning was mentioned in an ABPI trial using GammaPod^®^ [15,16]. In Canada, among 3894 patients who received unilateral whole breast radiotherapy at the Sunnybrook Health Sciences Centre in 2014–2018, only 80 patients (2.1%) were reported as treated in prone position [17]. Moreover, according to data collected from the Michigan Radiation Oncology Quality Consortium, prone RT was delivered in only 200 (4.3%) of 4688 breast cancer patients [18]. According to Australian data from a review in Brisbane, prone breast radiotherapy was reportedly offered to only 13 (1.8%) patients out of 708 patients treated in the supine position [19]. Thus, the odds are impressive. The purpose of this comprehensive review is to understand the reason for this paradox, encompassing a broad spectrum of prone positioning-related topics. It provides readers with effective insights into all the novel techniques and guidelines supporting this new approach as a good option in WBI.

## 2. Materials and Methods

A wide literature analysis was carried out using PubMed and EMBASE, applying keywords like ‘prone positioning radiotherapy’, ‘prone setup’, ‘prone breast radiation’, ‘prone breast and lymph node irradiation’, ‘prone position versus supine position’, and ‘prone DIBH’. The retrieved papers included only English articles focused on prone breast irradiation, referring to well-reported retrospective, observational phase II and randomized phase III studies; systematic reviews; and meta-analyses. In this search, all studies conducted within the last 25 years were included to provide readers with the main clinical scenarios needing an alternative treatment position to supine and show them how all criticisms related to this positioning have been responded to. The sequence of arguments were organized according several questions, as detailed below.

Which modalities and couches are appropriate for prone: dive or crawl? Are there real advantages in prone positioning in large size breast and left sided breast cancer? If supine DIBH is the best solution in sparing the heart and lungs in supine position, are there differences between supine DIBH (or otherwise voluntary inspiration breath hold) and free prone RT? Does DIBH in prone position add advantages in coverage of targets like nodal areas and in sparing OARs? If yes, is it reproducible? What about the effects of heavy particles in prone position? Is prone positioning useful in right-sided breast cancer?

In answer to these questions, we considered all clinical studies that refer to dosimetric experiences, patient’s quality of life reports, or safety data on prone positioning with and without nodal irradiation during conventional or hypofractionated radiotherapy with or without heavy particles. The literature search, screening of studies’ eligibility, data extraction, and database completion were performed independently by two investigators (G.L and C.M), and any discrepancies were resolved by consensus and, if necessary, by the opinion of a third reviewer (E.I.). Significant data are represented in a specific table. A visual depiction of different prone modalities and differences in dose distribution is provided in a synthesis of figures and pictures. No automation tool was used during the literature search.

## 3. Positioning Customized Immobilisation Systems

### 3.1. The Down Gravitational Dose Shift Effect in Prone Position

**Figure 1:**
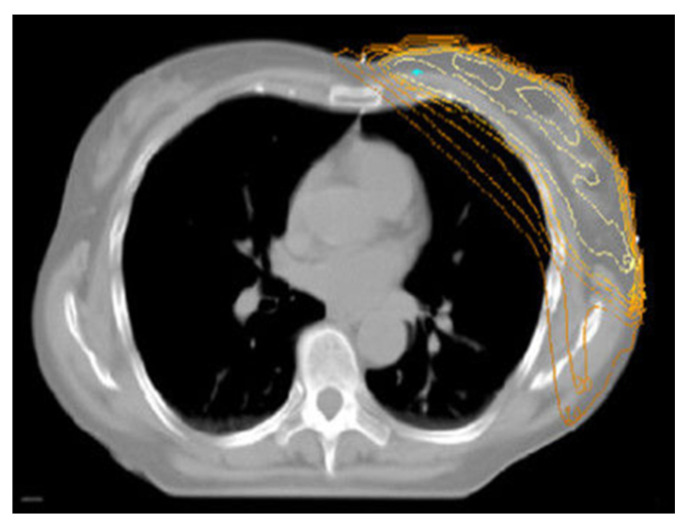
The lung and heart are covered by dose distribution across an irradiated breast in supine decubitus.

**Figure 2:**
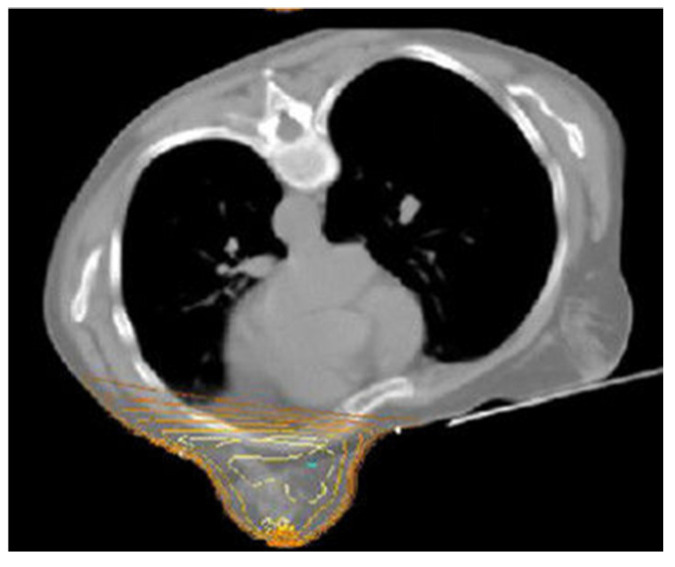
Down doses shift due to gravitational effect on heart and lung un-capped breast in prone decubitus.

**Figure 3:**
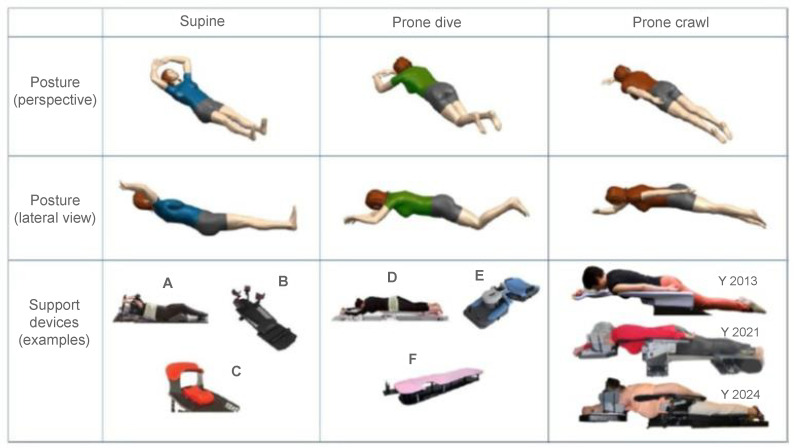
The figure presents the different positions: supine, prone dive, and prone crawl with different treatment supports. A-B-C for supine breast board; D-E-F for prone dive (F is the first prototype); 2013- and 2021-2024 for new prone crawl devices.

When comparing the dose distribution in supine vs. prone WBI, the feature that immediately catches the eye is the role of gravitational forces within the anatomical down shift of the breast and the chest, which makes a very important difference in toxicity. In supine WBI, the breast-capped lung and heart, the intramammary skin fold, and the spread-out breast are encompassed; in prone WBI, there is an uncapped lung and heart, outstretched skin folds, and elongated breast, leading to superior dose distribution; less skin reaction and cosmetic changes; and a much lower dose to the lung and probably to the heart (Figure 1 and Figure 2). Since the 1990s, a substantial number of trials have been conducted (mostly in the USA) involving several series of BC patient to assess the advantages of this gravitational dose shift in terms of dosimetry and toxicity when using prone position rather than supine WBI. The first studies focused on cases of large and pendulous breasts and left-sided breast cancer to resolve the effects of inhomogeneous dose deposition causing hotspot deposition and breast fold inflammation. In this regard, Bieri et al. previously conducted a simulation study on an anthropometric phantom treated in the supine and prone position, reporting significantly reduced integral lung, bone marrow, and cardiac doses with prone tangential fields [20]. Among pioneering clinical studies assessing the impact of dose hotspots, Merchant et al. reported a reduction in dose inhomogeneity by approximately 15% in cases of tangent fields adopted in women with pendulous breasts irradiated in the prone position compared to supine position [6]. In large-breasted women, the high-dose region calculated at the base of the breast ranged from 102 to 103% in the prone position instead of 116–118% obtained in the supine position. Identical dosimetric advantages were confirmed by a retrospective analysis by Mckinnon et al. The authors recorded homogeneous coverage of breast tissue with the prescribed dose, while the very few high-dose regions were displaced at the posterior medial and lateral borders and close to the most anterior point of the breast. Hotspots did not exceed 113% of the prescribed dose, with a maximum average of 107.6% [21]. In turn, the study of Grann et al. reported a remarkable cosmetic score outcome related to this effect. Furthermore, results on local control and survival appeared to be similar to those expected with conventional supine tangential fields [22]. Given this background, Stegman et al. conducted a retrospective study on 254 early BC patients treated with prone WBI. Initially, the protocol included only patients with large pendulous breasts; subsequently, patients affected by left breast cancer with significant comorbidities, extensive use of tobacco, and by patient or physician preference were also enrolled [23]. Dosimetric analysis was not performed; however, no worsening of such comorbid diseases was observed, and nor was radiation-induced pneumonitis reported. Lastly, specific studies conducted by Formenti et al. have contributed considerably to showing how gravity in prone position improves dosimetry. Data on 91 treated patients revealed the feasibility of achieving OAR sparing according to a protocol for the heart. As constraints, ILL should receive ≥18 Gy to 5% and ≥20 Gy to ≤10% of volume. These prescriptions were all met [24,25]. All these studies have been carried out using several different customized prone immobilization devices, which have evolved over time to implement two different prone conditions: the dive and crawl position (Figure 3).

### 3.2. Prototypes for the Prone Dive Position

Dive position is the most common modality when carrying out prone WBI on a customized device; the arms are blocked ahead, the legs are on cushioned wedges, and the treated breast hangs down through an open window in the table. The first prototype of prone-breast board was tested at the Memorial Sloan-Kettering Cancer Center (MSKCC) by Merchant et al. using a customized flat or curved table to be mounted on a simulator couch and LINAC table [6]. The device consisted of a hole in a wooden platform top through which the treated breast was hanging along the chest wall with the medial and lateral borders of the breast included within the field of the tangential parallel-opposed beams. The ipsilateral arm was placed at the side or above the head. At the University of Southern California (USC), Formenti et al. reported their first experience using a customized platform to adopt a radiosurgery-like technique for a partial breast irradiation in the prone position [24]. The board was a home-built solid wood platform containing a complex of removable concentrical inserts at the level of the breast, including several concentrical rings to insert breasts of different sizes. In the NY study, this device was fitted for whole breast prone irradiation to ensure a precise, reproducible, and comfortable setup. The side bar of the USC prototype was removed. The arms were up-sided, and, to improve the patient’s comfort, a prone mattress made of thick memory foam was used [25]. This complex ensured reproducibility through an equal degree of compression by the patient’s weight. This study also provided several solutions for a comfortable and more reproducible setup, such as a cushion wedge to push away the controlateral breast. In the trial by Stegman, a prone board with adjustable aperture and handgrip with a Styrofoam wedge for the contralateral breast was tested, receiving positive feedback. Mckinnon et al. also developed a system consisting of two 20 cm Styrofoam blocks through which the treated breast had to be suspended with a gap of approximately 15 cm between the two blocks. The arms were placed forward and the head in prone dive position, and the head was turned to the most comfortable side. To ensure more comfort and stability, a kneefix cushion was placed under the ankles [23]. Another solution for prone dive was proposed by Lakosi et al. This system satisfied patient comfort and reported data on residual-intrafractional errors in prone positioning with daily cone-beam computed tomography (CBCT). It consisted of three components: in the cranial part, the head was resting on a forehead–chin bracket, with the arms braced at the elbows and hands gripping a pair of handlebars. In the middle part of the thoracic portion, the treated breast was suspended freely through a cut-out while the contralateral thorax was supported by a horizontal board. The caudal portion was designed to support the pelvis and lower extremities with thermoplastic mask fixation. The mean overall reported comfort score was good as reported by patients, while the staff’s scores were generally lower [26].

### 3.3. The Prone Crawl Couch

The prone crawl couch is a novelty because it provides open access to the breast and II axillary levels, with the breast hanging in the vacuum space and no arm interposition. In comparison with prone dive, this position enhances the patient’s comfort and permits access to respiratory orifices, as demonstrated in patients cured in Intensive Care [27]. Furthermore, it delivers a less scattered dose due the easy access to the treated breast using smaller beam apertures and more separation between breast and OARs. As a result, it has been found that in patients requiring breast radiotherapy only, prone crawl position leads to a reduction of acute toxicity and cosmetic changes consisting of 70–80% reduction in lung dose, more than a 2% reduction in long-term lung cancer risk, and at least a 15% reduction in heart dose in left-sided RT [28]. In patients requiring breast and lymph node radiotherapy, its advantages have been estimated as nearly a 40% reduction in lung dose, a 20% reduction in heart dose in left-sided RT, and a 20% reduction in contralateral breast dose [29]. Researchers have investigated the comfort and effectiveness of this solution that reproduces the crawl swimming position of the arms: one arm is on the treated side alongside the body, and the other arm is on the contralateral side above the head supported by a new comfortable prone immobilization device: the crawl coach (CrC). This device is a novelty because it allows the irradiation of the breast, chest wall, and regional lymph nodes while blocking the controlateral breast using a customized red bra with black strips on laterals to fix the green alignment laser [29]. Compared with the dive, the crawl position has been validated for better patient comfortability, positioning stability, and setup precision. Importantly, it permits a wide range of beam directions in the coronal and near-sagittal planes with IMRT and VMAT modalities to cover the breast and regional lymph nodes without collisions with patients. Moreover, parts of the LINAC have a setup time (SUT) equal to time spent with the ordinary dive device [30]. Comfort and patient’s preference were assessed by a questionnaire administered at the time of simulation to each patient treated in dive and in crawl position [31], providing a score on the continuous visual–analogue scale to evaluate the pressure/tension and pain on several anatomical points. As a result, when asked to indicate which support device they preferred, 90% of the simulated patients preferred the crawl couch. Thus, this novel technique has been adopted in their institution routinely.

## 4. Advantages of Prone vs. Supine Position WBI

### 4.1. Breast Size and Acute Toxicity

Acute and late skin toxicity in women with large breast sizes irradiated in supine decubitus is a significant concern, owing to the hotspot deposition in inframammary fold and irregular thickness of breast parenchyma. All studies conducted on prone-position WBI have revealed an improvement in dose homogeneity, resulting in a reduced rate of acute skin toxicity in large and pendulous breasts as in dive as in crawl position. In regard to the dive position, in the study of Griems [32] conducted on 15 patients with different breast sizes, when comparing the two positions, the authors recorded a significant dosimetric advantage consisting of a reduction in the breast volume receiving more than 105% of the prescribed dose in the prone test. Furthermore, the homogeneity dose distribution was improved regardless of breast size (pendulous or otherwise). This effect resulted in low skin toxicity, while in a similar study by Mckinnon, as an acute toxicity outcome, desquamation developed in 50% of patients [21].

In a study by Kurtman et al. comparing supine and prone plans in five patients with moderate-to-large breasts, the decrease in hotspots was confirmed in four patients [33]. As a consequence of this better dose homogeneity, limited G3–G4 acute and chronic toxicities were observed in this series. In the study by Stegman, the rate of acute G2–G3 dermatitis was 16% and 2%, respectively. Meanwhile, the rate of chronic G2–G3 dermatitis at two years follow-up was 2.8% and 1.6%. To note, all patients included in this series had larger breasts. However, the observed rates of chronic toxicity were similar to data reported in the literature regarding women with an equal average breast size treated with supine WBI and conventional fractionation. Similarly, Vesprini et al.—in a phase3 multicenter single-blind randomized trial on prone vs. supine position in 378 women with large breast sizes (bra band ≥ 40 in, and/or ≥ D cup)—made similar conclusions. The desquamation rate, as the most monitored feature, was higher in the group of patients treated in the supine position (*p*  =  0.002) and then confirmed by multivariable analysis (OR, 1.99; 95% CI, 1.48–2.66; *p*  < 0.001). Other independent factors related to this variable were the use of boost (OR, 2.71; 95% CI, 1.95–3.77; *p*  <  0.001), the extended fractionation (OR, 2.85; 95% CI, 1.41–5.79; *p*  =  0.004), and bra size (OR, 2.56; 95% CI, 1.50–4.37; *p*  <  0.001) [34]. Looking to studies on crawl prone positioning, a low toxicity score on the LENT-SOMA scale has also been confirmed by a longitudinal analysis. As a result, for grade 2 toxicity, prone positioning resulted in a lower overall toxicity than that for supine positioning (*p* = 0.005) [30]. In the overview of the five-year photographic assessment, no significant difference in worsened breast cosmesis was observed between the groups at five years after radiation therapy [31].

### 4.2. Left-Sided Breast Prone Positioning Irradiation

Owing to concerns that radiation in the supine position is ‘more damaging to the heart’ in left-sided BC, as shown by Darby et al. [6], several attempts at prone versus supine decubitus have been made in the dive and crawl positions to demonstrate its usefulness in sparing heart in left-sided WBI [35]. However, not all of them reached a strong agreement. Griem et al. performed a dosimetric analysis comparing dose volume histograms (DVHs) for homogeneous target coverage, ipsilateral breast and contralateral breast volumes, ipsilateral and controlateral lung volumes, and heart volumes in 15 patients studied in supine and prone positioning (seven right and eight left) and with different breast sizes. Conformal 3D RT with tangential wedged photons beams was delivered to the breast. As a result, the volume of breast receiving > 5% of the prescribed dose was significantly lower in the prone position (*p* = 0.0074). In particular, the percentage volumes of lungs receiving ≥ 10 Gy and ≥20 Gy were significantly higher in the supine position for all the patients with left-sided lesions (*p* = 0.0001), although no differences were found for the integral dose to the contralateral breast (*p* = 0.6072) and the V30 Gy and V20 Gy of the heart (*p* = 1.000) [32]. Of course, the explanation for these unforeseen values is related to the anterior displacement of heart in the chest occurring in prone decubitus; this shift shortens the distance between the heart and the chest wall. Now, the measurements of this shortening due to this down anatomic displacement has been quantified by Chino et al. [36]. They compared the distances of the heart from the chest wall on the prone MRI with CT of the chest in the supine positions from 16 patients treated for left BC in free breathing. For each case, the distances between the heart and chest wall at nine points in three axial levels of the sternum in both supine CT and prone MRI were calculated. Using this analysis, a systematic displacement of the lateral and superior border of the heart closer to the chest wall in the prone vs. supine position was observed. The mean displacement was 19 mm (95% confidence interval 13.7–25.1 mm, *p* < 0.001). Meanwhile, no differences were recorded in the medial and inferior locations, which remained fixed. It is noteworthy that a reduction in volume of the lung within the heart and chest wall in the prone series was obtained with a mean decrease of 22 mL (*p* < 0.001 for significant difference). It should be noted that this finding could be critical in cases of a surgical bed located near the chest wall or deep in the breast. Recently, positive observations have been provided by a retrospective analysis conducted on 524 left-sided BC patients treated with moderate hypofractionated radiotherapy in the prone position. The results of this study were compared with literature data from supine treatments using the same hypofractionated schedule. The study verified the impact of prone positioning in minimizing the dose to the heart and LADCA. It identified the dosimetric parameter differences converted into EQD2 in terms of mean values (±SD) for MHD = 0.69 Gy (±0.19), LADCA D_mean_ = 2.20 Gy (±0.68), and LADCA D_max_ = 4.44 Gy 41 (±1.82) [37].

Regarding crawl prone positioning studies, all the comparisons done with supine revealed that the target volume coverages were not different between both positions; however, in prone position, the dose to OARs was significantly lower (*p* < 0.05) for the ipsilateral lung, contralateral lung, thyroid, esophagus. and skin, while there were no significant differences in heart and humeral head doses [29,30,31].

### 4.3. Supine-Free Breathing (S-FB) Versus Prone-Free Breathing (P-FB)

As reported above, in prone positioning, heart displacement down to the chest yields an uncertain advantage for heart dosimetry in left-sided breast radiotherapy within the tangential fields. The difference between S-FB and P-FB breathing has been assessed by a meta-analysis including 751 patients from 19 observational studies. Using the comparison between S-FB and P-FB, a significant advantage in OAR sparing was observed in P-FB concerning heart dose in terms of D_mean_ (*p* < 0.00009), D_max_ (*p* < 0.00001), V5 Gy and V20 Gy (*p* = 0.001), and LADCA D_mean_ (*p* = 0.005), D_max_ (*p* = 0.03), and V40 (*p* = 0.01). For the lung, similar outcomes were recorded (*p* < 0.00001) for ILL D_mean_, D_max_, V5 Gy, and V20 Gy in favor of P-FB. However, the differences in target coverage between the S-FB and P-FB groups were not statistically significant (*p* = 0.66) [38].

### 4.4. Supine Breath Hold Versus Prone Free Breathing

A randomized study—the UK Heart Spare Study—comparing voluntary supine deep inspiration breath hold (VBH) versus the free-breathing prone technique was conducted by Bartlett et al. to assess cardiac dosimetry differences in 34 enrolled patients who had an estimated breast volume of >750 cm^3^ requiring only WBI. Tangential fields were applied, and a moderate hypofractionation schedule of 40 Gy in 15 fractions was delivered. Patients were simulated in dive position on an Orfit AIO Solution ^®^ prone breast board and were randomized to receive the prone or supine VBH technique for the first seven fractions before switching techniques in the last part of the treatment. As a result, median WBCTV was similar for both techniques: 1064 cm^3^ (prone) vs. 1029 cm^3^ (VBH). The difference between WBCTV for prone and VBH radiotherapy plans in all patients was < 0.001). All heart dosimetric parameters were statistically better in the supine VBH than in prone treatment. In supine VBH, the Heart NTD_mean_ was 0.44 [0.38–0.51] vs. 0.66 [0.61–0.71] (*p* < 0.001); LADCA NTD_mean_ was 2.9 [1.8–3.9] vs. 7.8 [6.4–9.2] (*p* < 0.001), respectively; and LADCA D_max_ was 21.0 [15.8–26.2] vs. 36.8 [35.2–38.4]. Only lung dosimetry improved in prone position in terms of ipsilateral and whole lung NTD_mean_ than in supine VBH, at 0.34 [0.27–0.42] vs. 3.7 3 [3.42–4.04] (*p* < 0.001) and 0.20 [0.16–0.24] vs. 1.81 [1.65–1.97] (*p* < 0.001), respectively. The mean controlateral breast dose was lower in VBH than in prone, at 0.10 [0.08–0.11] vs. 0.33 [0.23–0.43] (*p* < 0.001). The mean CB dose was significantly lower with VBH than prone treatment, at 0.10 [0.08–0.11] vs. 0.33 [0.23–0.43] (*p* < 0.001). Displacements errors and systematic moves were predominant in prone while treatment setup times and total treatment session times were significantly less with VBH (*p* = 0.01, *p* = 0.002 respectively). The study was stopped for the following reasons: better cardiac dosimetry with VBH techniques, failure to meet inclusion criteria, prone treatment plan issues, prone equipment shortcomings, and prone setup difficulties. The authors concluded that supine VBH is advantageous and time-sparing [39].

## 5. RT Advanced Techniques with DIBH in Prone Positioning

### 5.1. Prone DIBH Experiences

**Figure 4:**
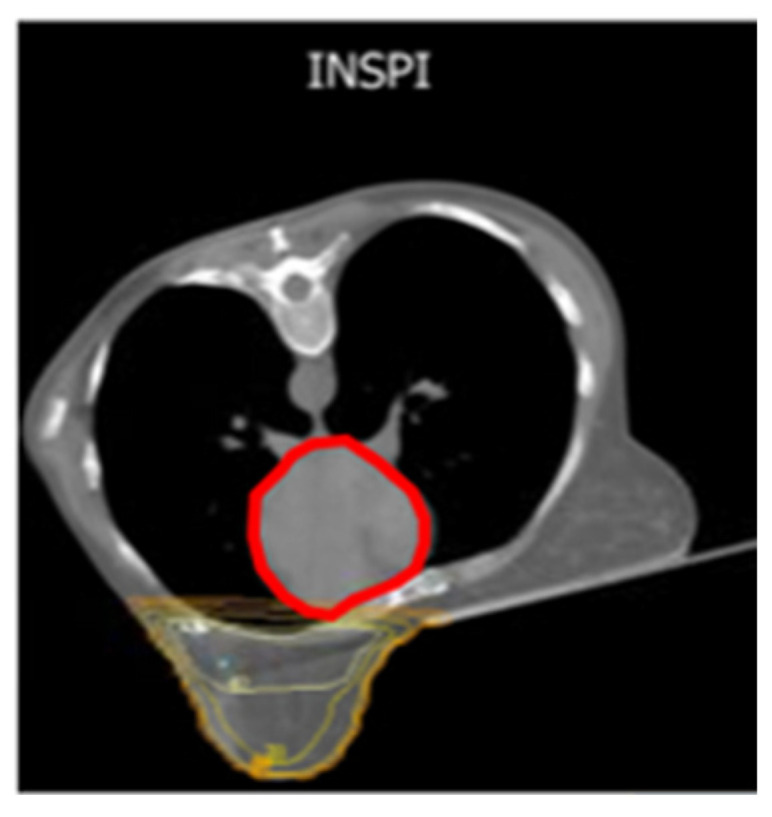
Dose distribution to heart in inspiration (in red the position of heart down in the chest wall).

**Figure 5:**
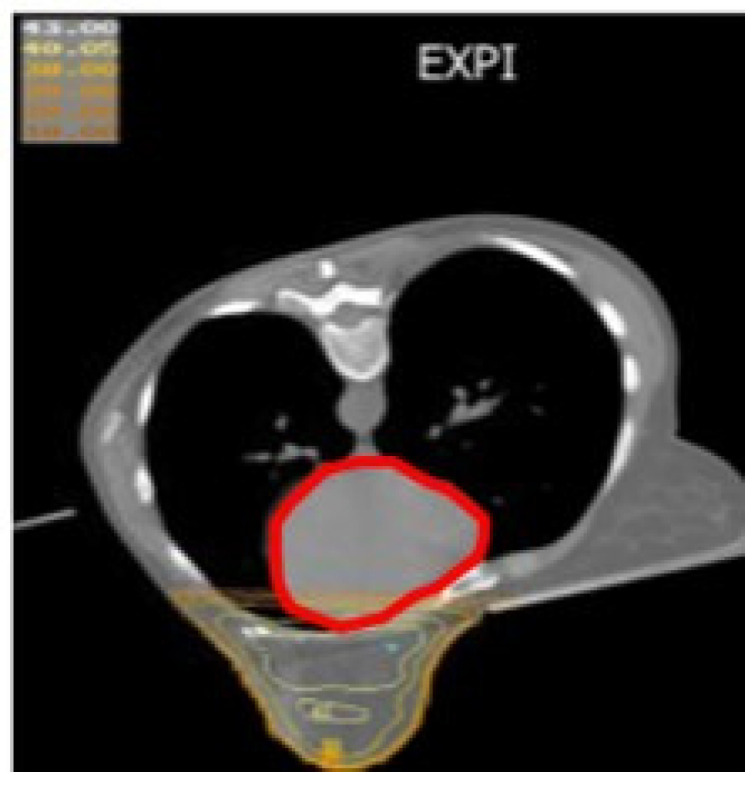
Dose distribution to heart in expiration (in red the position of heart down in the chest wall).

Given the well-acknowledged role of DIBH in reducing the dose to the heart, LADCA, and ILL in left-sided BC in supine treatments regardless of fractionation schedules [40], it is reasonable to provide this modality in prone positioning, as depicted in Figure 4 and Figure 5. Thus, prone DIBH combining the gravitational effect on anatomy with the increased distance between the heart and breast may address the problem by reducing the dose to the heart in this position. In the previous meta-analysis, the comparison between P-FB and S-DIBH was reported only within two studies [38]. In the study of Mulliez et al., the S-DIBH resulted in a lower dose to the heart (*p* < 0.001), while in the P-FB, the doses to the LADCA and ILL were statistically (*p* < 0.001) minimized [41]. In the study by Saini et al., no significant differences in heart and LADCA dose between the two groups were observed, while in P-FB, a significant reduction in the dose to the ILL was found (*p* < 0.001). Finally, when comparing P-DIBH with S-DIBH, a dosimetric advantage was demonstrated in doses to the heart, LADCA, and ILL (*p* < 0.001) for P-DIBH in both studies. Although the LADCA dose did not reach significant statistical difference in Saini’s study, its value in P-DIBH was lower than in S-DIBH [42].

In a first dosimetric report by Mulliez on DIBH in prone position for left-sided WBI, the authors were able to demonstrate the efficacy of this technique in minimizing the dose to the heart in left-sided BC patients treated with whole breast irradiation and moderate hypofractionation. Twelve patients were enrolled and underwent simulated CT scans in supine shallow breathing (SB), supine DIBH, prone SB, and prone DIBH. A validation cohort was provided, with 38 patients receiving prone SB and prone DIBH CT-scans. The final 30 patients were subject to prone DIBH treatment. As a result, the dose coverage index did not differ significantly: 91.1 ± 3.3% for supine SB (explorative group), 91.2 ± 3.7% for supine DIBH (explorative group), 93.3 ± 3.7% for prone SB (all patients), and 93.0 ± 3.9% for prone DIBH (all patients). Then, a validation trial was conducted to assess the heart dose/volume results provided by the exploratory study, taking into account, as a planning goal, a D_max_ < 5 Gy and D_mean_ < 1 Gy to the contralateral breast for all positions and different breast volumes. Thus, reductions in heart D_mean_ in prone DIBH compared to prone SB were recorded as follows: for breast volume less than 750 cc (18 patients) = 1.3 (±0.9 Gy); between 750 and 1500 cc (22 patients) = 0.7 (±0.7 Gy); and larger than 1500 cc (10 patients) = 0.4 (±0.4 Gy). Moreover, the LADCA D_mean,_ D_max_ and lung doses were lower. A following trial on 51 early-stage left-sided BC patients treated with WBI after breast-conserving surgery was performed with CT simulation in prone SB and DIBH [41]. Through the rigid co-registration of a prone-positioned patient in SB and DIBH, the matching showed quantitative volume variations, centroid shifts, elongations, and overlap indices for the ipsilateral breast, clips, IMLN, heart, and lungs, all in favor of prone DIBH vs. SB. In prone DIBH, the heart volume was decreased by 4.3%, thus achieving a medial, posterior, and caudal centroid heart shift. According to the breast–heart distance, in prone DIBH, the volume of the heart within smaller distances to the breast was reduced, assuming a statistically significance (*p* < 0.05) for the breast [43].

### 5.2. Deep Inspiration BH in Crawl Prone Position

A specific study was conducted to validate the dosimetric advantages of DIBH in the crawl position vs. prone shallow breathing (SB). Thirty-one patients with invasive carcinoma and node positive of the left breast were included in this study [44]. Photon and proton plans were carried out and then compared. The patients were positioned on the prone crawl breast couch as described above. DIBH activity was monitored using magnetic sensors placed on the surface of the breast couch and lateral thoracic wall. The patients underwent two CT scans for radiotherapy planning, first in a short DIBH of approximately 15 s and then in SB. PTV also included axillary-level II–IV lymph nodes and the ipsilateral mammary internal (MI) lymph nodes. For photon plan optimization, a non-coplanar multiple overlying short-arc VMAT technique was used in order to achieve optimal beam directions and a lower dose given to OARs. The dose objectives were reached for all the targets in all the plans. Compared with SB, the DIBH technique significantly decreased the mean dose to the heart in both photon and proton plans. The mean heart dose reduction in DIBH (compared with SB) for photon and proton plans was on average 2.0 Gy (range: −1.0–3.5) and 0.56 Gy RBE (range: 0.1–1.1), respectively. DIBH also resulted in a significantly lower mean dose for the esophagus for photon plans, but not for protons. On average, in the photon plans, the left lung mean dose decreased by approximately 13% with the use of DIBH. Meanwhile, in proton DIBH plans, the average mean left lung dose increased by approximately 21%. No significant differences were observed for proton or photon plans in terms of mean dose to the contralateral breast [44].

## 6. Reproducibility in DIBH Prone Positioning

Reproducibility in repeated DIBH is a challenging issue in supine as in prone positioning. In the study by Muellez, 21 over 30 consecutive female left-sided BC patients were irradiated only on the breast with prone positioning on a modified prone BB in SB and DIBH. The patient’s breathing motion was registered as described above. During simulation, one prone SB and two prone DIBH CT scans (DIBH1-DIBH2) were acquired. The first DIBH scan (DIBH1) was the reference for treatment purposes. The second scan (DIBH2) was used to verify the anatomical reproducibility of DIBH in the prone position. Delineation of the heart, both breasts, and lungs was performed in SB, DIBH1, and DIBH2. A rigid registration of the DIBH1 and DIBH2 CT scans was conducted to evaluate the anatomical reproducibility of the DIBH phase. The overlap index was calculated for the ipsilateral breast, heart, and both the lungs. The overlap index was defined as the intersection of the volumes on DIBH1 (VDIBH1) and DIBH2 (VDIBH2) divided by the volume on DIBH1 (VDIBH1): the higher result in the anatomical reproducibility was assumed to be the overlap index. The average and standard deviation of the DIBH magnitude and intra-breath hold instability were calculated for each treatment session to evaluate the intra-fraction DIBH reproducibility and instability. As a result, the population range of the DIBH was four times larger than that of the SB. This effect revealed the capability of patients to perform and maintain a repeated deep breath in the prone position. In fact, the intra-fraction standard deviation of the DIBH size was 1.0 ± 0.4 mm (range 0.5–1.9 mm) in confirmation of the high reproducibility of breath hold amplitudes during one treatment fraction. The number of breath holds required to deliver the treatment ranged from four to seven, each lasting on average 16 ± 1 s. This resulted in a treatment time of 300 ± 69 s (range 231–445 s) [45]. Deseyne et al. tested the feasibility and intra-fraction reproducibility of the repeated breath hold technique (RBH) in the prone position in cases of WB with RNI after breast-conserving surgery using the prone crawl couch. Each patient underwent a free-breathing CT scan and a breath hold scan as well as an additional low-dose RBH scan to evaluate the positional and dosimetric impact of RBH and failure to breath hold (FTBH). The RBH test was monitored using two magnetic sensors as described above. The whole breast, axillary levels II–IV (LNN II-IV), and mammary internal nodes (LNN MI) were the specific targets (TVs). As a result, a significant absolute volume difference between BH and FTBH scans was recorded for the contralateral breast, heart, lungs, and level II (*p* < 0.05), while there were no differences in dose coverages in all the TVs for RBH. In FTBH, there were numerically significant but clinically less relevant dosimetric differences for CTV WBI, LNN III, and LNN IV, while larger differences were consistent in D95 and D98 for LNN II and LNN MI. With regard to OARs, there were no relevant dosimetric differences for RBH with the exception of V30 to the thyroid. In FTBH, there were significant dose differences for all the OARs except for the thyroid and right lung [46].

## 7. Nodal RT in Prone Position

### 7.1. Ghent University Group Guidelines

New guidelines have been developed to define the nodal targets in prone positioning. This group started from the existing ESTRO14 and PROCAB13 guidelines for supine positioning and went on to define new guidelines for prone radiotherapy considering the anatomical differences between the two positions. Practically, to detail the method, 18 MRI and 9 CT datasets were evaluated to include inter-patient anatomical variability. The MRI scans in the supine position (SP) and prone crawl position (PCP) were used to indicate reference structures and delineate nodal targets according to the ESTRO and PROCAB supine position guidelines. During the process of selecting new reference structures, the resulting PCP CTVs were compared routinely with the SP CTVs to ensure that similar structures (mainly the veins) had to be contained within the CTVs (steps 2 and 3). As novelties, the cranial and dorsal border of level IV; the lateral border of level III; and the cranial, lateral, ventral and dorsal borders of level I were adapted from the ESTRO and PROCAB guidelines to obtain PCP specific guidelines. These new PCP-specific guidelines incorporated the anatomical variability between patients and are now always applicable [47].

### 7.2. New York Langone University Guidelines

This group conducted an adaptation of the RTOG BC delineation guidelines from supine to prone position through an evaluation by two radiation oncologists and a breast radiologist. The anatomic variations from supine to prone position in 43 patient-representative cases treated in the prone position were analyzed. Level I and II axillae were defined on simulation CT scans compared with preoperative diagnostic supine imaging. The resulting contours were reviewed by an expanded expert multidisciplinary panel to achieve a consensus in creating an atlas specifically for CT scan nodal delineation in the prone position. For level II, the cranial, posterior, lateral, and medial borders remained unaltered. Meanwhile, the anterior and caudal borders were revised to better include the intrapectoral and subpectoral nodes, up to where the axillary vessels cross the lateral edge of the pectoralis minor muscle. For level I, the cranio-caudal borders remained unaltered, whereas the other borders were revised due to difference in positions of landmarks between those of supine and prone positions [48].

### 7.3. Regional Lymph Node Irradiation

Coverage of axillary lymph nodes in prone position is another challenging issue, as assessed by Haffty in a review [49]. Several studies have verified if nodal irradiation in prone positioning could be feasible and effective, but a preliminary experience conducted by Alonso-Basanta et al. in a group of 20 breast cancer patients did not show favorable dosimetric coverage of these volumes. In this study, patients underwent two CT simulations (in the supine and prone positions) to assess the possibility of covering nodal targets and sparing the normal tissues using a standard tangential technique. As a result, in both positions, treatment of the nodal regions was not possible. In fact, the mean dose to the nodal regions for levels I-III was 50% less in the prone position as compared with the supine position, although there was better OAR sparing in the prone position. The mean ipsilateral lung volume receiving 95% of the prescribed dose was 6.3% in the supine position compared to 0.43% in the prone position. The mean heart volume receiving 30 Gy was 0.56% in supine compared with 0.30% in the prone position, suggesting a higher risk of nodal recurrence with the prone setup in cases of nodal irradiation [50].

A first prospective trial of radiation therapy to the breast, postmastectomy chest wall, and supraclavicular and level III axillary lymph nodes in prone decubitus was conducted on 69 patients with stage IB-IIIA BC by Shin et al. A dose of 40.5 Gy/15 fractions with a concomitant daily boost to the tumour bed of 0.5 Gy (total dose 48 Gy) was prescribed using 3-IMRT fields. A dosimetric comparison between prone protocol and non-protocol conventional three-field/four-field supine plans was performed to evaluate the dose to the heart and lungs. As a result, the coverage of the breast/tumor bed and chest wall was within the required dose constraints in all cases. This approach revealed significantly decreased lung V10 Gy and heart V5 Gy doses in prone plans. The V38.5 Gy prescription to PTVnodes ≥ 95% was achieved in the overwhelming majority (except in 10% of cases). The main acute toxicity was G1 radiation dermatitis, which occurred in almost all treated patients. Meanwhile, none experienced grade 2 acute skin toxicity. Grade 1 lymphedema was the most common side effect, and an excellent/good score for cosmesis was obtained [51].

A phase 1/2 trial for stage IB-IIA BC was performed by Purswuani et al. on 97 patients using a hybrid 3D-IMRT technique and moderate hypofractionated radiotherapy in the prone position after lumpectomy or mastectomy with and without inclusion of nodal areas. The target volume coverage and normal tissue goals were reached in almost all cases. In fact, in prone plans, V48 Gy ≥ 98% was achieved in 92% of cases, breast V40.5 Gy ≥ 95% was recorded in 98% of the patients, and the nodal V38.5 Gy ≥ 95% was obtained in 89% of the cases. The constraints on the OARs were also respected. No acute toxicity over G2 was recorded. After 8 years of median follow-up, grade 2–3 late toxicity was recorded in 23% of the treated patients. No chest wall or nodal recurrence was observed in the subsequent follow-up [52].

It is also important that we report data from a first retrospective test study conducted by Speleers et al. In this test, ten left-sided BC patients with invasive carcinoma of the breast with positive lymph nodes requiring nodal RT were enrolled. Six patients were simulated in the supine and prone crawl positions, while four patients were scanned in prone crawl position. As target volumes, the breast (WBI), the II-III axillary level (AxII-III), the inter-pectoral, peri-clavicular (PC), and internal mammary (IM) lymph node areas were considered. Nodal delineation was performed according to the guidelines above. Plans were formulated using a supine coplanar or prone non-coplanar multiple overlying partial-arc VMAT technique using photons and protons. A median dose of 40.05 Gy (GyRBE) in 15 fractions was prescribed to PTV-WBI, PTV-PC, and PTV-IM. The aim was to obtain 95% of the volumes covered with at least 95% of the prescribed dose (i.e., 38 Gy) and for no more than 5% to receive 105% of the prescribed dose (i.e., 42 Gy). A comparison study between protons and photons was conducted, but no significant differences were observed between the photon and proton plans with regard to the maximum PTV-WBI and PTV-PC doses. Comparable maximum and higher minimum target doses resulted in better dose homogeneity for the proton plans than for the photon plans in the supine and prone crawl positions. The mean doses to the OARs were lower for prone crawl than for the supine positions and for proton than for photon plans. The lowest average mean thyroid dose was obtained in the prone crawl photon plans, which showed statistically significant results versus all the other plans. The lowest average mean and D02 esophageal dose was obtained in the supine proton and supine photon plans, respectively. The coverage of the breast and nodal targets was similar in the supine and prone crawl positions. However, the minimum dose in the proton plans was higher for all the targets, leading to better dose homogeneity than in photon plans for the supine and prone crawl positions [53].

## 8. Heavy-Particle RT Modalities in Supine vs. Prone Positioning

First experiences in prone positioning with protons have been described above using only IMRT-VMAT modalities [44]. However, more complex techniques with heavy particles—such as intensity-modulated proton therapy (IMPT), proton arc therapy (PAT), intensity-modulated carbon-ion therapy (IMCT), and carbon arc therapy (CAT)— have been tested and have expanded treatment possibilities beyond conventional rays (XRT). Heavy-particle modalities such as proton (PBT) and carbon-ion radiation (CIR) treatments have been implemented to minimize dose deposition outside the target area and allow for enhanced dosimetry with IMRT techniques.

A pioneering study was conducted by Kim et al. The authors evaluated how different RT modalities could influence dosimetry in terms of the target and OAR outcomes in supine versus prone positioning WBI and mitigate the long-term side effects of radiation. They attempted to assess differences in the dose distribution among plans with six external beam WBI techniques (3D-CRT, VMAT, IMPT, PAT, IMCT, and CAT) in terms of the treatment position, breast size, the heart’s proximity to the breast, and risks associated with secondary cancer. Fourteen patients were enrolled and underwent simulated free breathing in supine and prone positioning. The radiotherapy plans were evaluated with respect to the homogeneity index (HI) and Paddick conformity index (PCI) of the PTV. In the prone-position evaluations, the PTV’s PCI was superior to that in the supine position for all the studied techniques. However, in the supine position, a statistically significant reduction in the mean heart dose was recorded for most treatment techniques. Furthermore, in the context of XRT, the prone position showed a reduced mean lung dose and reduced V20 values for the ipsilateral lung. In contrast, for PBT, these values were minimal regardless of the patient’s position. In terms of the separation between the PTV and OARs, in the supine position, a more substantial separation effect for the heart was achieved. Meanwhile, in the prone position, the separation effect of the ipsilateral lung from the PTV was not satisfactorily achieved when compared with the supine position (*p* < 0.05). Secondary cancer risk estimation was quantified by a parameter defined as EAR (excess absolute risk) for an individual exposed to radiation at a dose (D) at age (e) according to Schneider’s OED concept (organ-equivalent dose for radiation-induced cancer). As a result, PBT in the supine but not prone position showed a smaller EAR than CIR for the lungs and controlateral breast [54].

## 9. Prone Positioning in Right-Sided Breast Irradiation

If prone WBI is advantageous in left breast RT, it is reasonable to apply the same advantages for right-sided breast RT in terms of the lowest radiation dose to non-target organs, regardless of breast size. Prone-position radiotherapy for right-sided BC should be less difficult because the distance between the tangential field’s edge and the heart is less influenced by the gantry perspective on the breast. Fargier-Bochaton et al. assessed this issue in a retrospective study on 146 patients with right BC treated in the prone position after a simulation with supine positioning. A penalty score was computed from the mean absolute dose deviation to the heart, lungs, breasts, and tumour bed for each patient’s supine and prone plans. The dosimetric advantage of prone positioning was assessed via the reduction in penalty score from supine to prone position. Compared with supine position, in prone, the penalty score was reduced in 119 patients (81.5%). The lung doses were also reduced from 4.8 Gy in supine to 1.4 Gy in prone position. Prone position was associated with a dosimetric advantage in most patients. Thus, the option to be treated in the prone position should be offered to right-sided BC patients [55].

## 10. Prone APBI

Trials with high-quality evidence have shown the safety and effectiveness of APBI and its lack of inferiority to WBI; examples include the Florence trial [56] and IMPORT LOW [57]. Thus, prone APBI seems to be a promising modality, but CTV delineation is a challenging issue, and the use of breast MRI images could be helpful [58]. There are still uncertainties that need to be solved, like tumor bed delineation on the basis of clip locations and the safe range of margins to be added. A comparison with preoperative CT may help to show adequate expansion margins within which to improve accuracy and achieve a good balance between reduced dose and volume in order to comply with APBI rationale [8]. Discussions of this topic will appear in future research.

## 11. Discussion

Given the assumption that there is no evidence that one positioning technique is superior to another, supine treatment remains the standard positioning in WBI, while the DIBH modality has proven to be the best system with which to reduce the dose to the heart [40]. However, valid alternative treatment positioning has been tested to address advantages in dose homogeneity, cosmesis, and low skin toxicity in patients with large pendulous breasts for reasons other than reducing the dose to the heart in left-sided breast irradiation. Prone decubitus has attracted growing interest over the past 20 years. As reported by Haffty, when conducting a search on PubMed of the 10 years before 2008, only a few reports (at least 20) concerning prone breast irradiation could be identified. Meanwhile, over 100 reports published from 2008 to 2018 could be found [49]. This implies that prone positioning continues to be a highly intriguing topic for investigation [58], as shown by its adoption in APBI recently. The advantage of prone radiotherapy is due to the gravitational dose shift effect as a consequence of the anatomical shift of the treated breast hanging far down from lung and heart, which leads to better dose homogeneity mainly in pendulous large breasts. In response to the commentary by Haffty, reasonable criticisms have been leveraged regarding uncertainties in setup reproducibility, the true advantages of OAR sparing, and coverage of targets including nodal irradiation; however, novel literature data show the solution of these issues and support the advantages of this positioning with advanced techniques. Prone positioning has been demonstrated to achieve better dosimetry in large and pendulous breasts mainly in obese women, wherein supine treatment could be challenging. Acute and chronic toxicity have been shown to be similar to or better than that in the supine position, regardless of breast size and fractionation. Customized immobilization coaches have evolved to improve patients’ treatment comfort and setup reproducibility. The position of the arms has been shifted from a dive position to a novel comfortable one similar to the swimming crawl position, which has been shown to allow IMRT delivery with a wide range of beam directions to cover the breast and regional lymph nodes without collisions with the immobilization device, regardless of photon or proton use [6,7,8,9,10]. Regarding nodal irradiation, several attempts have been conducted in dive as well as in crawl position [14,30,31]. The axillary levels and chest wall are now well targeted in prone decubitus, with good dosimetric coverage and definition by a customized atlas with validated contouring guidelines [47,48]. However, the best achievement has been made in dosimetry for the heart and LADCA, owing to experiences with prone DIBH, which have defied the odds [43,44,45,46]. A summary is shown in Table 1.

## 12. Conclusions

Convincing novel evidence on the advantages of WBI in the prone position has been provided, showing that prone DIBH is dosimetrically more advantageous than SB for OARs. In particular, the prone crawl position has been shown to be more convenient than the dive prone position in terms of patient comfort and IMRT beam arrangements that permit nodal irradiation coverage without collisions with the patient and LINAC components. Heavy particles could be considered for better dosimetry and low toxicity. Misconceptions regarding prone positioning as a time-consuming modality have also been dispelled. Undoubtedly, complete agreement is needed among the radiation team for collaboration at many stages in order to address all the challenges related to prone irradiation radiotherapy; DIBH procedures should combine patients’ comfort and confidence with precise setup on customized immobilization devices. Prone APBI is another issue under investigation with promising results [57]. Despite these advances, there is a paradox between the growing evidence of benefits and the weak clinical use of prone treatment worldwide, because supine treatment seems more available, and thus it remains the gold-standard treatment. Better knowledge of these novel procedures and widespread sharing of this literature data could help to overcome this paradox.

## Figures and Tables

**Table 1:** The most important topics and literature data supporting the prone position.

***Topic***	***Author/Year***	***Technique/Fractionation***	***Main Outcomes of Prone***
***Large and pendulous breasts***	Merchant, IJROBP, 1994 [6]	Conventional fractionation, 2D	High-dose regions reduced to 102–103% in the prone position vs. supine.
	Grann, IJROBP, 2000 [22]	Conventional fractionation, 3D	Skin erythema and Grade I or Grade II breast edema. The mean overall cosmesis score was 9.37 (range, 8–10).
	Kurtman, Braz J Med Biol Res, 2003 [33]	Conventional fractionation, 3D	Mean RT doses to ILL 8.3 ± 3.6 Gy in the supine position vs. **1.4 ± 1.0 Gy in the prone position (*p* = 0.043)**. The heart values were 4.6 ± 1.6 and 3.0 ± 1.7 Gy (*p* = 0.079).
	Formenti, JCO, 2007 [25]	Hypofractionation, IMRT	5% of heart volume to receive ≥18 Gy, and ≤10% of ILL volume to receive ≥20 Gy; this was achieved in all patients.
	Mckinnon, Breast, 2009 [21]	Conventional 3D fractionation	Grade 3 acute dermatitis and edema occurred in 2% of patients. Chronic Grade 2 to 3 skin and subcutaneous tissue toxicities were reported in 4.4% and 13.7% of patients.
	Vesprini, JAMA, 2022 [34]	Conventional 3D fractionation Hypofractionation	Desquamation in the supine position compared with prone was 39.6% vs. 26.9% (*p* = 0.002).
***Topic***	***Author/Year***	***Technique/Fractionation***	***Main Outcomes of Prone***
***Advantages in*** ***left-sided BC***	Griem, IJROBP, 2003 [32]	Conventional fractionation, 2D	The average volume of lung receiving >10 Gy and >20 Gy was significantly less in the prone positions. (*p* = 0.0001). The integral dose delivered to the contralateral breast was not significantly different (*p* = 0.6072). Heart V30 Gy and V20 Gy no advantage.
	Deseyne, Sci Rep, 2017 [14]	Hypofractionation, IMRT, Crawl position	Reduction in doses (*p* < 0.05) to ipsilateral lung, contralateral breast, thyroid, esophagus, and skin. No significant differences for heart and humeral head doses.
	Lai, 2021 Medicine [38]	Meta-analysis	Advantages in P-FB for heart: D_mean_ (*p* < 0.00009), D_max_ (*p* < 0.00001), V5 and V20 (*p* = 0.001), LADCA D_mean_ (*p* = 0.005), D_max_ (*p* = 0.03), V40 (*p* = 0.01). For lung: ILL D_mean,,_ D_max,,_ V5 and V20 (*p* < 0.00001).
	Gregucci, Cancers, 2025 [37]	Hypofractionation, IMRT	Reduced doses to heart and LADCA in EQD2 in terms of mean values (±SD) for MHD = 0.69 Gy (±0.19); LAD D_mean_ = 2.20 Gy (±0.68); LAD D_max_ = 4.44 Gy 41 (±1.82).

## Data Availability

Data are available in the References section.

## Abbreviations

APBI	Accelerated Partial Breast Irradiation
AxII-III	Axillary levels II-III
BB	Breast Board
BC	Breast Cancer
CAT	Carbon Arc Therapy
CIR	Carbon-Ion Radiation
CBCT	Cone Beam Computed Tomography
CrC	Crawl Coach
CTV	Clinical Target Volume
D02-D95-D98	Dose to a percent value of organ volume
3D-CRT	Three Dimensional Conformal RT
DIBH	Deep Inspiration Breath Hold
D_max_	Maximum Dose
D_mean_	Mean Dose
DVHs	Dose–Volume Histograms
EAR	Excess Absolute Risk
EQD2	Equivalent Dose in 2 Gy fractions
ESTRO14	European Society for Radiotherapy and Oncology14
FTBH	Failure To Breath Hold
HI	Homogeneity Index
ILL	Ipsilateral Lung
IMCT	Intensity-Modulated Carbon-Ion Therapy
IMLN	Internal Mammary Lymph Node
IMPT	Intensity-Modulated Proton Therapy
IMRT	Intensity-Modulated Radiation Therapy
LADCA	Left Anterior Descending Coronary Artery
LENT-SOMA scale	Late Effects on Normal Tissue—Subjective, Objective Management and Analytic scale
LINAC	LInear ACcellerator
LNN	Axillary Lymph Node level
MHD	Mean Heart Dose
MI	Ipsilateral Mammary Internal nodes
MRI	Magnetic Resonance Imaging
NTDmean	Biologically Weighted Mean Of Total Dose to Tissue Normalized to 2 Gy Fractions Using a Standard Linear Quadratic Model
OARs	Organs at Risk
OED	Organ-Equivalent Dose for Radiation-Induced Cancer
PAT	Proton Arc Therapy
PBT	Proton Beam Therapy
PC	Periclavicular nodes
PCI	Paddick Conformity Index
PCP	Prone Crawl Position
P- DIBH	Prone Deep Inspiration Breath Hold
P-FB	Prone Free Breathing
PROCAB	PROject on CAncer of the Breast
PTV	Planning Target Volume
RBE	Relative Biological Effectiveness
RBH	Repeated Breath Hold
RNI	Regional Node Irradiation
RT	Radiotherapy
RTOG	Radiation Therapy Oncology Group
SB	Shallow Breathing
S- DIBH	Supine Deep Inspiration Breath Hold
S-FB	Supine Free Breathing
SP	Supine Position
SUT	Setup Time
TVs	Target Volumes
V5-20-30- 38.5-40 Gy	Percent of Organ Volume Receiving 5 Gy, 20 Gy, 30 Gy, 38.5 Gy-40 Gy
VBH	Voluntary Breath Hold
VDIBH1/VDIBH2	Intersection of the Volumes on DIBH
VMAT	Volumetric Modulated Arc Therapy
WBI	Whole Breast Irradiation
WBCTV	Whole Breast Clinical Target Volume
XRT	Conventional Rays
